# Paroxetine ameliorates prodromal emotional dysfunction and late-onset memory deficit in Alzheimer’s disease mice

**DOI:** 10.1186/s40035-020-00194-2

**Published:** 2020-05-12

**Authors:** Peng-Hui Ai, Si Chen, Xian-Dong Liu, Xiao-Na Zhu, Yuan-Bo Pan, Dong-Fu Feng, Shengdi Chen, Nan-Jie Xu, Suya Sun

**Affiliations:** 1 Department of Neurology and Institute of Neurology, Rui-jin Hospital Shanghai Jiao Tong University School of Medicine, Shanghai, 200025 China; 2 Collaborative Innovation Center for Brain Science, Department of Anatomy and Physiology, Shanghai Jiao Tong University School of Medicine, Shanghai, 200025 China; 3 Department of Neurosurgery, Shanghai Ninth People’s Hospital, Shanghai Jiao Tong University School of Medicine, Shanghai, 200025 China; 4 Key Laboratory of Cell Differentiation and Apoptosis of the Chinese Ministry of Education, Shanghai Jiao Tong University School of Medicine, Shanghai, 200025 China; 5 Shanghai Key Laboratory of Reproductive Medicine, Shanghai Jiao Tong University School of Medicine, Shanghai, 200025 China

**Keywords:** Alzheimer’s disease, Paroxetine treatment, Glutamate receptor, Memory deficit

## Abstract

**Background:**

Neuropsychiatric symptoms (NPS) such as depression, anxiety, apathy, and irritability occur in prodromal phases of clinical Alzheimer’s disease (AD), which might be an increased risk for later developing AD. Here we treated young APP/PS1 AD model mice prophylactically with serotonin-selective re-uptake inhibitor (SSRI) paroxetine and investigated the protective role of anti-depressant agent in emotional abnormalities and cognitive defects during disease progress.

**Methods:**

To investigate the protective role of paroxetine in emotional abnormalities and cognitive defects during disease progress, we performed emotional behaviors of 3 months old APP/PS1 mouse following oral administration of paroxetine prophylactically starting at 1 month of age. Next, we tested the cognitive, biochemical and pathological, effects of long term administration of paroxetine at 6 months old.

**Results:**

Our results showed that AD mice displayed emotional dysfunction in the early stage. Prophylactic administration of paroxetine ameliorated the initial emotional abnormalities and preserved the eventual memory function in AD mice.

**Conclusion:**

Our data indicate that prophylactic administration of paroxetine ameliorates the emotional dysfunction and memory deficit in AD mice. These neuroprotective effects are attributable to functional restoration of glutamate receptor (GluN2A) in AD mice.

## Background

Alzheimer’s disease (AD) is the most common form of dementia and the most prevalent neurodegenerative disease characterized by cognitive disorder and memory dysfunction in the elderly population, affecting almost 40 million people worldwide [1, 2]. Neuropathologically, AD is accompanied by synaptic loss, deposition of Aβ plaques, neurofibrillary tangles (NFTs), and hyperphosphorylated tau [3]. These changes are developed progressively which ultimately results in AD pathology in the late period [4, 5].

Although these changes can be found in some AD patients and animal models, clinical trials have failed to give an effective treatment to prevent, halt, or reverse AD. Due to the irreversible development of AD, it appears to be critical to diagnose and intervene in the early stage. AD is now known to have a long preclinical phase before the onset of clinical symptoms [6]. Recent studies have shown that neuropsychiatric symptoms (NPS) are strong features of AD and related dementia [7]. The symptoms of depression, anxiety, apathy, and irritability occur in prodromal phases of clinical disease [8]. Of all the NPS, depression and apathy are the most frequently observed symptoms in people with mild cognitive impairment (MCI) and early AD [9]. Moreover, mild behavioral impairment patients without cognitive symptoms are more likely to develop dementia [10, 11], and those with MCI progress to AD at a much higher rate if they have NPS [12], indicating a history of depression or anxiety confer an increased risk for later developing AD [13].

Serotonin-selective reuptake inhibitors are widely prescribed for the treatment of clinical depression and anxiety disorders [14], but may also have therapeutic potential as neuroprotective agents [15, 16]. Previous findings have reported that the treatment with serotonin-selective reuptake inhibitors might ameliorate some of the behavioral deficits found in aged APP/PS1 mice [17] and the levels of amyloid beta-peptide (Aβ) and numbers of Aβ are significantly reduced in the hippocampus of paroxetine-treated 3xTgAD mice [18]. Serotonin enhance synaptic plasticity by activating cyclic AMP response element-binding protein (CREB) and up-regulating the expression of brain-derived neurotrophic factor (BDNF) [19], and this signaling mechanism is compromised in AD [20]. There is evidence that antidepressant treatment increases expression of glutamate receptor subunit to enhance the synaptic plasticity which is impaired in the AD [21–23]. Thus, we aim to investigate how APP/PS1 mice behave in emotional state in the early stage, and if prophylactic administration of serotonin-selective reuptake inhibitor alleviates AD-like pathology and cognitive impairment in the AD mouse model.

In the present study, we find that the APP/PS1 mice display emotional dysfunction in 3 months. The long-term treatment of paroxetine ameliorates memory impairment of AD mice in late period, and this is might through restoring glutamate receptor subunit levels and function. Our results thus indicate therapeutic potential of prophylactic treatment with paroxetine for late cognitive defects in AD.

## Methods

### Mice

APP/PS1 mice (APPKM670/671NL, PS1L166P) [24] were kindly provided by Mathias Jucker (Tubingen University). All experiments involving mice were carried out in accordance with the US National Institutes of Health Guide for the Care and Use of Animals under an Institutional Animal Care and Use Committee approved protocol and Association for Assessment and Accreditation of Laboratory Animal Care approved Facility at the Shanghai Jiao Tong University School of Medicine. Parents and pups (10–11 pups per litter) were raised in animal facilities with a constant temperature (22 °C) and on a 12-h light-dark cycle. Food and water were unlimited to access. The day of birth was defined as postnatal day 0 (P0). All efforts were made to minimize the number of animals used and their suffering.

### Animal treatment

For prophylactic treatment, mice received paroxetine (15 mg/kg) or 0.9% saline starting at 1 month of age. Mice received paroxetine in their drinking water (0.1 mg/ml) and this water consumption was replaced every second day. The emotion state of young adult mice was evaluated at 3 months and the ability of memory was examined till 6 months.

### Immunofluorescence

For immunofluorescence, mice were anesthetized (chloral hydrate, 350 mg/kg), perfused transcardially with 0.1 M PBS followed by 4% paraformaldehyde in phosphate buffer. The brains were then removed, postfixed and sectioned at 30 μM in the coronal plane using a vibratome. Vibratome sections were blocked with permeable buffer (0.3% Triton X-100 in PBS) containing 10% donkey serum for half an hour at room temperature, incubated with 6E10 (1:500, Biolegend), anti-serotonin (1:500, Abcam) in permeable buffer containing 2% donkey serum overnight at 4 °C. The slices were then washed three times with PBS-T (0.1% Tween-20 in PBS) for 10 min every time, and incubated with Alexa Fluor secondary antibodies (1:200, Molecular Probes) and NeuroTrace 633 (1:500, Molecular Probes) in the PBS buffer for 2 h at room temperature. Slices were washed in PBS-T for three times and mounted on glass slides using Aqua poly/mount (Polysciences), and photographed using confocal microscope (Leica Application Suite X).

### Western blotting

For western blotting, the procedure was performed as previous study at the age of 6 months [25]. Analysis of the data was performed using NIH ImageJ software, and the mean density of each band was normalized to β-actin signal in the same sample and averaged. For primary antibodies, we used mouse anti-β-actin (1:3000, Thermo, MA5–15739), rabbit anti-Tau (phosphor-S396) (1:1000, Abcam, ab109390), mouse anti-GluA1 (1:1000, Santa Cruz, sc-13,152), goat anti-GluA2 (1:200, Santa Cruz, sc-7611), mouse anti-GluN1 (1:1000, BD, 556308), rabbit anti-GluN2A (1:1000, Millipore, ab1555P) mouse anti-GluN2B (1:1000, BD, 610417).

### Behavioral analyses

#### Open field

All tests were conducted according to previous study [26]. Three-month mice were habituated to handling and transported from the colony room to the behavioral room for 3 days before behavioral tests were begun. Mice were given 1 h to habituate after transport to the behavioral room before any tests were conducted. All apparatuses and testing chambers were cleaned with 75% ethyl alcohol wipes between animals.

The open field (40 cm × 40 cm × 40 cm) was used and adult mice were placed in the central area and recorded for 20 min. Locomotor activity was assessed as path length. All the tests were performed in a sound-attenuated and temperature-controlled (23 ± 1 °C) room illuminated by one 40-W fluorescent bulb placed 3 m above the apparatus. Digitized video recordings (30 frames /s) with EthoVision software (Noldus Information Technology, Leesburg, VA) were employed for behavioral analysis.

### Elevated plus maze

The EPM apparatus was made of dark grey plastic and consisted of two open arms (30 × 7 × 0.25 cm) opposed to two enclosed arms (30 × 7 × 15 cm) elevated 60 cm from the floor. Animals were placed in the central area of the apparatus with their head facing an enclosed arm (test duration: 5 min). The test was performed in a sound-attenuated and temperature-controlled (23 ± 1 °C) room illuminated by one 40-W fluorescent bulb placed 3 m above the apparatus. Digitized video recordings (30 frames per second) with EthoVision software (Noldus Information Technology, Leesburg, VA) were used for behavioural analysis.

### Forced swimming test

The Forced Swimming Test (FST) is typically used in rodents to screen for potential human antidepressants. In the FST, immobile time is used as a measure of depressive behavior. Three-month mice were placed into clear plastic buckets 20 cm in diameter and 23 cm deep filled 2/3 of the way with 22 °C water. The test was conducted for 2 consecutive days. For day 1, mice were exposed to the swim 5 min for habituation. For day 2, the test was conducted as day 1 described. Test was videotaped from the side for 5 min and the immobile time was calculated for the last 4 min.

### Three-chamber test

A Plexiglas cage was divided in three compartments. Both side compartments contained an empty perforated cup. For social behavior, firstly, the tested mouse was allowed to explore freely the whole setting, with all doors open for 5 min. After the habituation period, the mouse was restricted in the middle compartment, while an unfamiliar mouse of the same sex (stranger) was placed under one of the cups (sides alternated between each mouse). The tested mouse was then allowed to explore the whole apparatus for 5 min. The tested mouse could then again freely explore the whole apparatus for 5 min. In all these stages, time spent in each compartment and contact with the cup (close interaction) were automatically recorded. Social index was calculated by ratio of time interacting with stranger mouse to that with empty cup.

### Fear conditioning

Before test, 6-month mice were habituated to handling and given 1 h to habituate after transport to the behavioral room before any tests were conducted. On the first day, mice were placed in the conditioning boxes to explore freely for 20 min and then returned to its home cage. On the second day, mice were placed in the same conditioning boxes to explore freely for 3 min, and then a sound cue was played for 30s and immediately closed and an electric foot shock (0.85 mA, 2-s duration) was delivered through the floor grid. The mouse was taken out 30 s after termination of the foot shock and returned to its home cage. On the third day, mice were either placed in the same conditioning boxes without sound cue to explore freely for 3 min (context A), or novel conditioning boxes that completely different from the previous conditioning boxes to explore freely for 3 min and then the sound cue was played for 1 min (context B). Mice behavior were recorded by digital video cameras mounted above the conditioning chamber. FreezeFrame and FreezeView software (Ugo Basile, Italy) were used for recording and analyzing the freezing behavior, respectively.

### In vitro electrophysiology

The electrophysiology was conducted in the prefrontal cortex of 6-month mice. Brains were dissected quickly and chilled in ice-cold artificial cerebrospinal fluid (ACSF) containing: 125 mM NaCl, 2.5 mM KCl, 2 mM CaCl2, 1 mM MgCl2, 25 mM NaHCO3, 1.25 mM NaH2PO4, 12.5 mM Glucose with 95% O2 /5% CO2. Coronal brain slices (400 mm thick) were prepared with a vibratome and transferred to a chamber with bubbling with 95% O2 and 5% CO2 at 31 °C for 1 h and then maintained at room temperature (22–25 °C). Neurons were targeted for whole-cell patch-clamp recording with borosilicate glass electrodes having a resistance of 5–8 MΩ. The electrode internal solution was composed of 115 mM CsMeSO3, 10 mM HEPES, 2.5 mM MgCl2.6H2O, 20 mM CsCl2, 0.6 mM EGTA, 10 mM Na phosphocreatine, 0.4 mM Na-GTP and 4 mM Mg-ATP. To determine NMDAR-to-AMPAR ratio, peak amplitude of ESPCs at − 70 mV in presence of 100 μM picrotoxin was measured as AMPAR-mediated currents, and peak amplitude of EPSCs at + 40 mV in presence of 100 μM picrotoxin and 20 μM CNQX was measured as NMDAR mediated currents. For NMDAR subunit mediated currents, 3 μM ifenprodil or 0.4 μM NVP-AM007 (NVP) were applied in the bath solution. The decay time was measured with Clampfit.

### Bioinformatics analysis

GSE 39697 dataset was downloaded from GEO database of NCBI. The raw RNA expression profile datasets were preprocessed using R 3.4.1 statistical software together with a Bioconductor package. In accordance with the R package, the robust multiarray average (RMA) algorithm was used to adjust for background intensities in the Affymetrix array data by including optical noise and non-specific binding (NSB). The background adjusted probe intensities were then converted into expression measures using the normalization and summarization methods encapsulated by RMA algorithm. The k-Nearest Neighbor (KNN) algorithm was used to generate the missing values. We used the R-package, limma, to assess the differential expression genes (DEGs) between 0 day and 30 days of granular cell layer (GCL). DEGs were selected for which both the log2-FC values were greater than 0.5 and *P* values were < 0.05. We performed Gene Ontology (GO) enrichment analysis with the DEGs by functional annotation tools in Database for Annotation, Visualization and Integrated Discovery (DAVID). We compared the genes in the three GO terms and acquired 5 overlapped genes. Venn diagram was made by Venn diagram tool (http://bioinformatics.psb.ugent.be/webtools/Venn/).

### Statistical analysis

The results are presented as mean ± SEM were determined by Student’s t test for two-group comparisons or ANOVA followed by Sidak’s post hoc test for multiple comparisons among more than two groups.

## Results

### Paroxetine ameliorates emotional dysfunction in early-age APP/PS1 mice

**Fig. 1:**
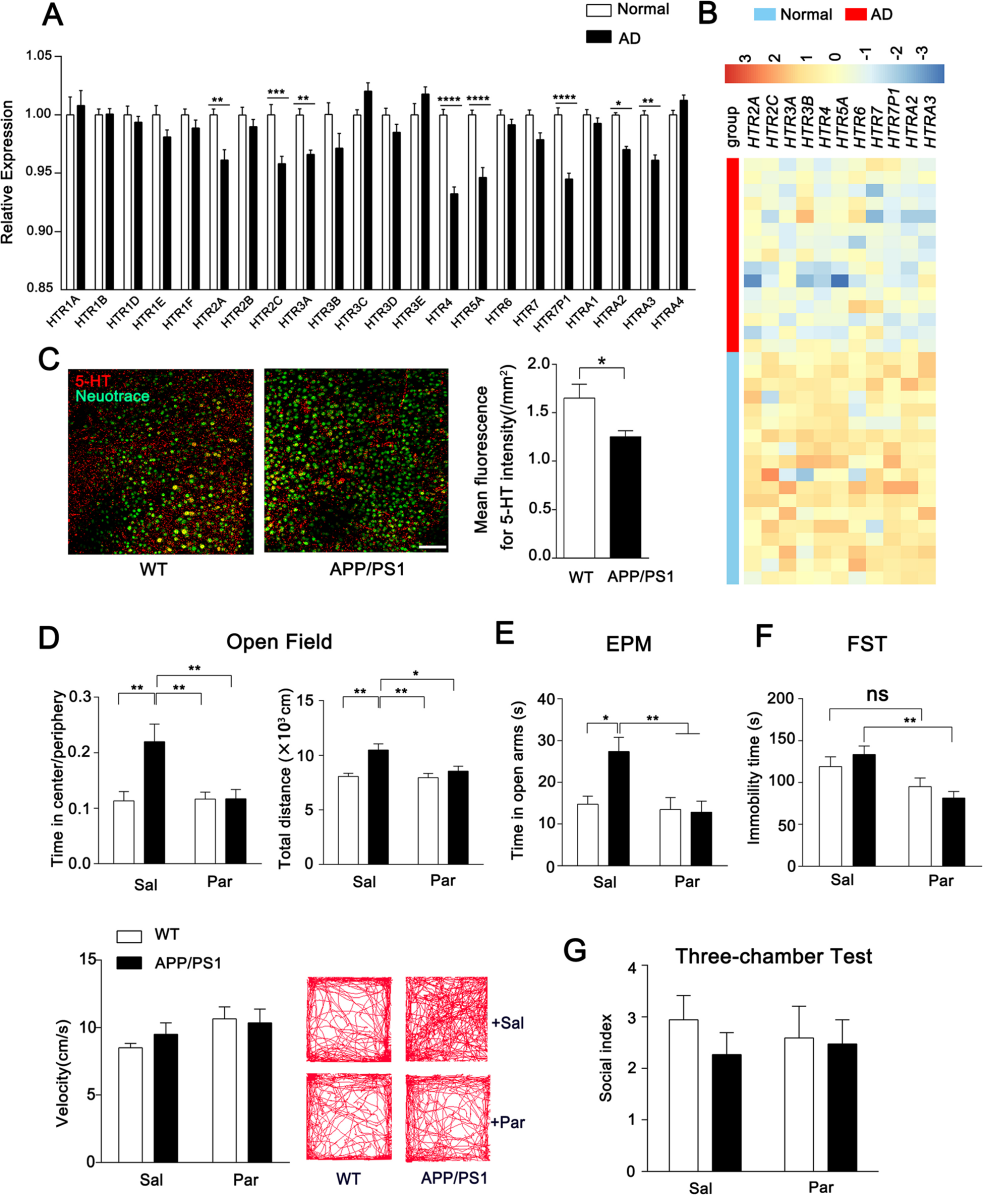
Paroxetine ameliorates emotional dysfunction of APP/PS1 mice in the early age. **a** Relative expression of 5-HTergic receptors for AD patients compared with normal acquired from transcriptional analysis. **b** Heatmap of significant decreased 5-HTergic receptors. **c** Representative Immunofluorescence of serotonin in the prefrontal cortex and quantification of 3-month WT and APP/PS1 mice. *n* = 8. **d** Percent of time in center of open field test and total movement distance for each group for saline or paroxetine treated WT and APP/PS1 mice (Upper panel). Velocity and representative track map for each group (Down panel). *n* = 11–13. **e** The time spent in the open arms of elevated plus maze for saline or paroxetine treated WT and APP/PS1 mice. *n* = 11–13. **f** Immobility time of Forced-swim test for saline or paroxetine treated WT and APP/PS1 mice. *n* = 10. **g** Social index of Three-chamber test for saline or paroxetine treated WT and APP/PS1 mice. *n* = 7–8. Data are presented as mean ± SEM. *P < 0.05. ** *P* < 0.01; *** *P* < 0.001; **** *P* < 0.0001; two-way ANOVA with Sidak’s multiple comparison post hoc test (**a**, **d**, **e**, **f**, **g**) and unpaired t-test (**c**)

At early stage of AD and related dementia, the symptoms of depression, anxiety, apathy, and irritability occur in prodromal phases of clinical disease. We performed transcriptional analysis of prefrontal cortex from AD patients to determine whether the expression of serotonergic (5-HTergic) system was changed compared with normal human. The result shows a certain extent decrease of the expression for 5-HTergic receptors (Fig. 1a, b). We also conduct immunofluorescence of serotonin in the prefrontal cortex and found a mild decrease of serotonin level in the APP/PS1 mice compared with WT mice (Fig. 1c).

Since the 5-HTergic system is involved in the regulation of mood, the amelioration of emotional dysfunction for early-age APP/PS1 mice may achieved by response of 5-HTergic receptors to SSRI. We firstly assessed anxiety using open-field test to evaluate emotion state of young adult mice at 3 months, which has been used widely for measure the anxiety for rodents [27, 28]. In the open-field test, the percentage of time spent in center was significantly increased for APP/PS1 mice compared with WT mice, suggesting occurrence of emotional dysfunction with low levels of anxiety, while the group of APP/PS1 mice following paroxetine treatment showed similar behaviors to WT mice. In addition, we also observed that APP/PS1 mice display more movement distance in open filed while the velocity of each group showed no difference (Fig. 1d). The anxiolytic phenotype was also evaluated by Elevated Plus Maze. The results showed that APP/PS1 mice spent longer time in open arms in comparison with WT mice (Fig. 1e). In addition to the anxiety, we also evaluated other behaviors such as depressive state with FST or social ability with three-chamber test in the early age of APP/PS1 mice. Although the immobility time of APP/PS1 mice in FST showed no significance compared with WT mice, paroxetine treatment caused a decreased immobility time in APP/PS1 mice, which confirms antidepressant-like effects of paroxetine (Fig. 1f). In the three-chamber test, those group showed no difference indicating normal social ability for APP/PS1 mice in the early age (Fig. 1g).

### The memory deficit and Aβ plaque are attenuated in paroxetine-treated APP/PS1 mice

**Fig. 2:**
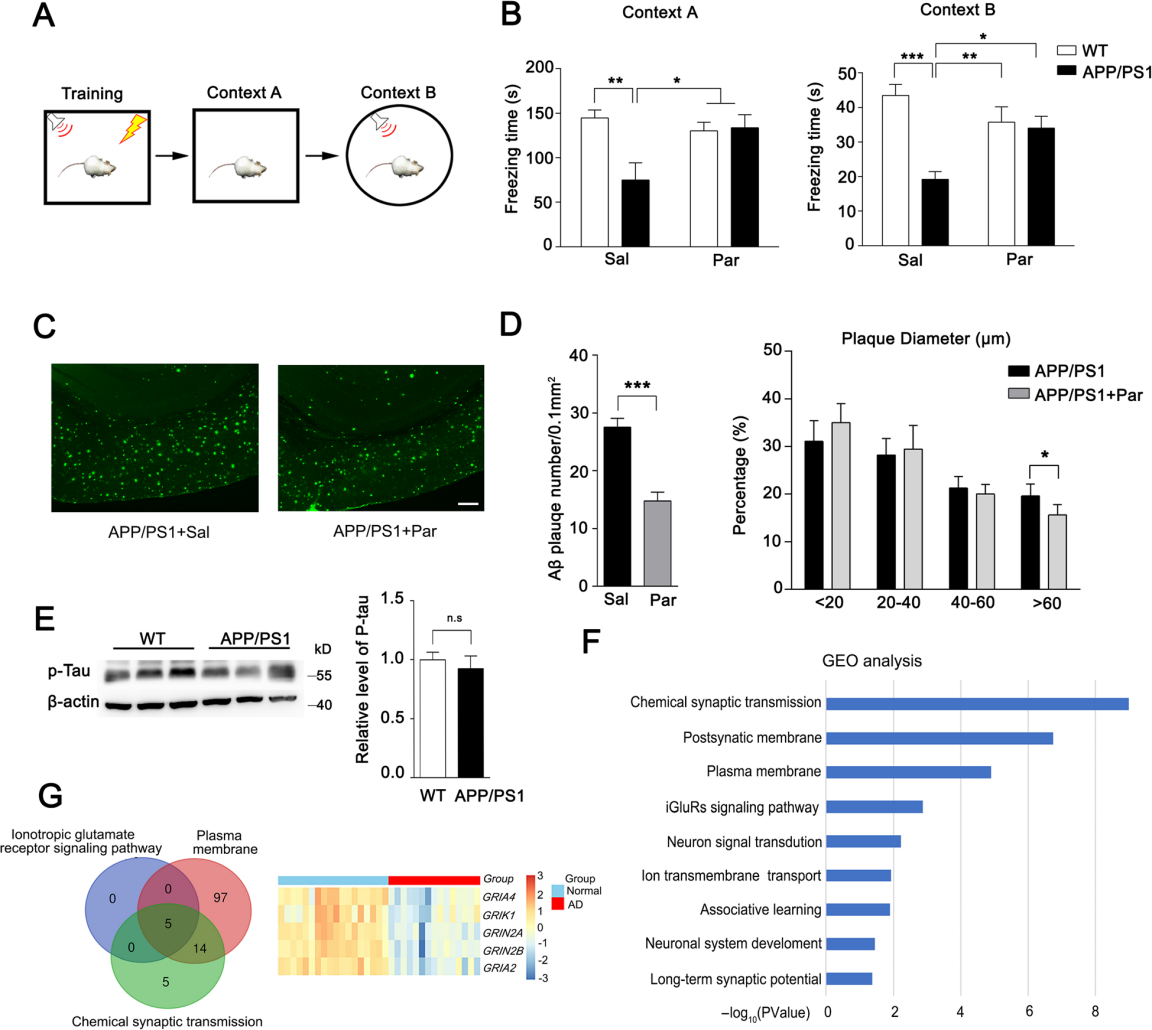
Paroxetine treatment attenuated memory deficit and Aβ accumulation of APP/PS1 mice in the late period. **a** Schematic of fear condition test. **b** Freezing time in context A and context B of FCT for saline or paroxetine treated WT and APP/PS1 mice. *n* = 9–11. **c** Representative immunohistochemical staining shows Aβ immunoreactivity in the cortex of APP/PS1 mice that treated with saline or paroxetine. Scale bar, 100 μm. **d** Results of quantitative analysis of Aβ-positive neurons in the cortex of APP/PS1 mice that had been treated with saline or paroxetine. *n* = 10. Data are presented as mean ± SEM. *P < 0.05; ** *P* < 0.01; *** *P* < 0.001; two-way ANOVA with Sidak’s multiple comparison post hoc test (**b**) and unpaired t-test (**d**). **e** Representative western of tau phosphorylation in the cortex of 6-month WT and APP/PS1 mice are shown and quantified *n* = 9. Data are presented as mean ± SEM and determined by Student’s t test. **f** Results of GO Enrichment analysis on the set of differentially expressed genes (DEGs). The length of each bar indicates the log10 (*P*-value) and the vertical axis shows significantly enriched terms. **g** Venn diagram of three GO term (Left panel). Overlapped 5 genes with differential expression were shown by heatmap (Right panel)

The main characteristic of AD is memory deficit. Considering paroxetine treatment in early age APP/PS1 mice abolishes the emotional dysfunction, as a high risk for AD pathogenesis, we asked whether the treatment is sufficient to rescue the memory deficit of AD mice in the late period. We conducted fear conditioning test at 6-month-old APP/PS1 mice following long-term paroxetine administration. As the results shown, the saline-treated APP/PS1 mice showed obvious memory deficit with a significantly decreased freezing time compared to saline-treated WT mice in both context A and context B test, while paroxetine treatment resulted in a significant restoration in freezing time comparable to the saline-treated AD mice (Fig. 2**a, b).** These results suggested that paroxetine early treatment is benefit for memory deficit rescue of AD mice. Given that the deposition of Aβ and phosphorylated tau mediated the neurofibrillary tangles are two main core pathological features of AD, we asked whether paroxetine treatment affected the accumulation of Aβ in AD mice, and evaluated Aβ immunoreactivity in brain sections from saline-treated and paroxetine-treated AD mice using antibody 6E10. We observed significantly decreased numbers of Aβ plaque in the cortex of paroxetine-treated mice compared with that of saline-treated AD mice. The percentage of Aβ plaque with large diameter (> 80 μm) was also obviously decreased for APP/PS1 mice after paroxetine treatment (Fig. 2c, d). No Aβ immunoreactivity was observed in brain sections from WT mice, which shows that paroxetine early treatment could decrease the deposition of Aβ plaque for AD mice. P-TAU did not show significant difference in 6-month-old APP/PS1 mice (Fig. 2e).

### Long-term paroxetine regulates glutamate receptor function

**Fig. 3:**
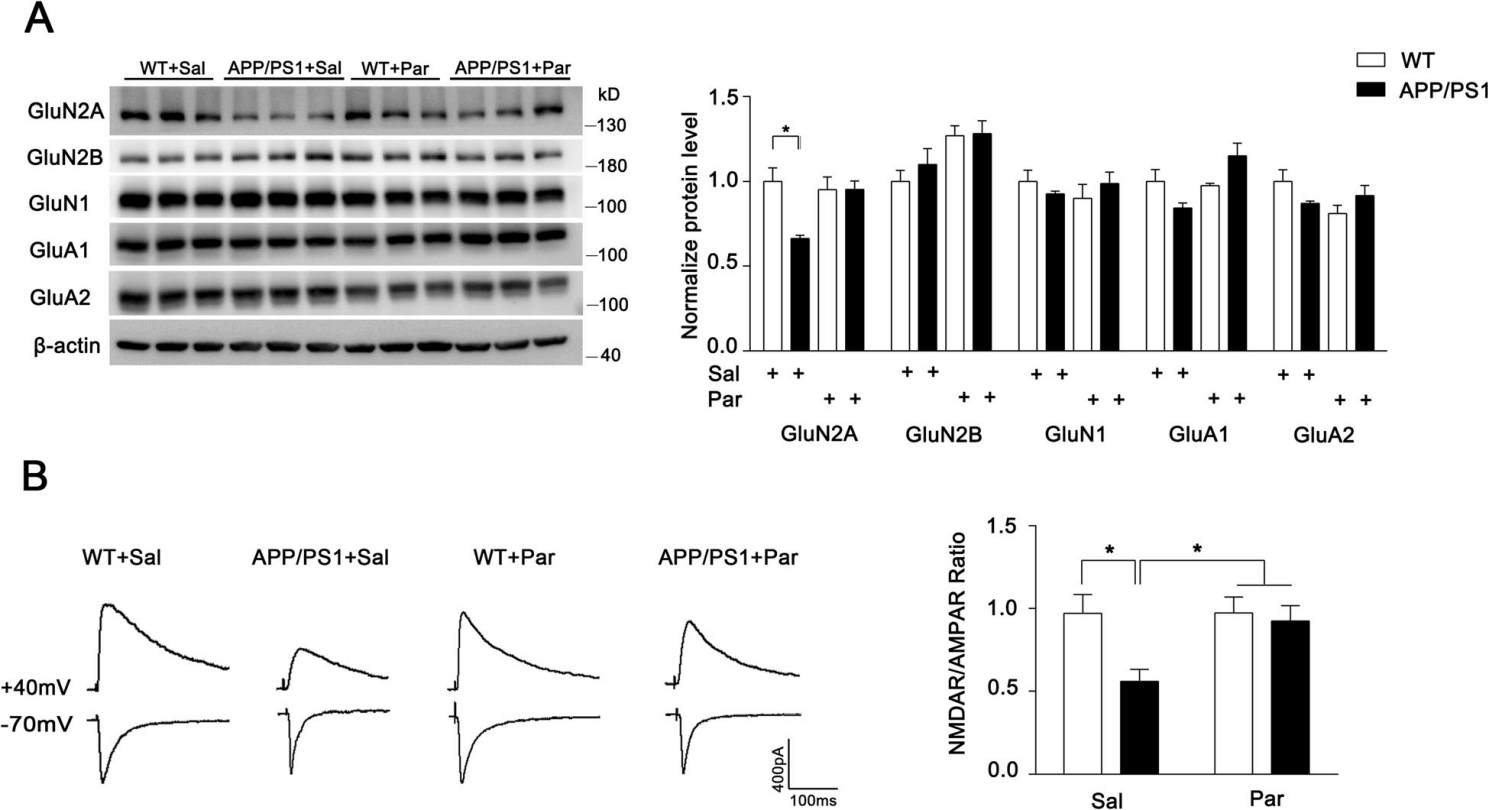
Paroxetine treatment restores glutamate receptor subunit of GluN2A levels and NMDAR function decreased in AD mice. **a** WBs of glutamate receptor subunits (GluN2A, GluN2B, GluN1, GluA1 and GluA2) in cortical homogenates are shown. The left panel shows representative western blots and the right panel shows quantification of WBs. *n* = 3 mice for per group. Data are presented as mean ± SEM. * *P* < 0.05; unpaired t-test. **b** Representative traces of AMPAR EPSCs recorded at − 70 mV and NMDAR EPSC at + 40 mV. Scale bar: 400pA (vertical) × 100 ms (horizontal). *n* = 9–16 neurons from 3 to 5 mice for per group (left panel). Ratio of NMDAR/AMPAR in cortex was quantified respectively for per group (Right panel). Data are presented as mean ± SEM. * *P* < 0.05; two-way ANOVA with Sidak’s multiple comparison post hoc test

To investigate the mechanism of memory deficit for AD mice, we performed transcriptional analysis of prefrontal cortex from AD patients to determine the differential expressed genes (DEGs) compared with normal human and further conducted GO analysis for the DEGs. These changed genes for AD patients were significantly enriched in GO terms of plasma membrane and chemical synaptic transmission, suggesting that synaptic ion channels may largely affected in AD patients (Fig. 2f). Therefore, we compared the three GO terms and observed 5 overlapped genes. Of the 39 DEGs, the glutamate receptors gene *GRIA4*, *GRIK1*, *GRIN2A*, *GRIN2B* and *GRIA2* are majorly involved (Fig. 2g). As the well-balanced two key glutamate receptor subtypes for synaptic function, NMDAR and AMPAR are strongly required for memory formation [29, 30]. To investigate the mechanism of memory rescue for paroxetine treated APP/PS1 mice, we next measured the NMDAR and AMPAR level, and found an obviously reduced GluN2A expression in APP/PS1 mice compared to WT mice, while no significant change was observed in other glutamate receptor subunits. After long-term paroxetine treatment, the level of GluN2A was largely restored (Fig. 3a). Thus, long-term paroxetine treatment seems to induce a change of glutamate receptor subunit with higher ratio of NMDAR to AMPAR for APP/PS1 mice. To confirm the results of biochemical analysis, we conducted electrophysiology to measure the NMDAR/AMPAR ratio directly. Consistent with the previous western result, saline-treated APP/PS1 mice displayed a significant decrease of NMDAR/AMPAR ratio compared to saline-treated WT mice and the altered ratio was restored to normal level after the treatment of paroxetine (Fig. 3b).

### The functional up-regulation of NMDAR subunit GluN2A after long-term paroxetine treatment

**Fig. 4:**
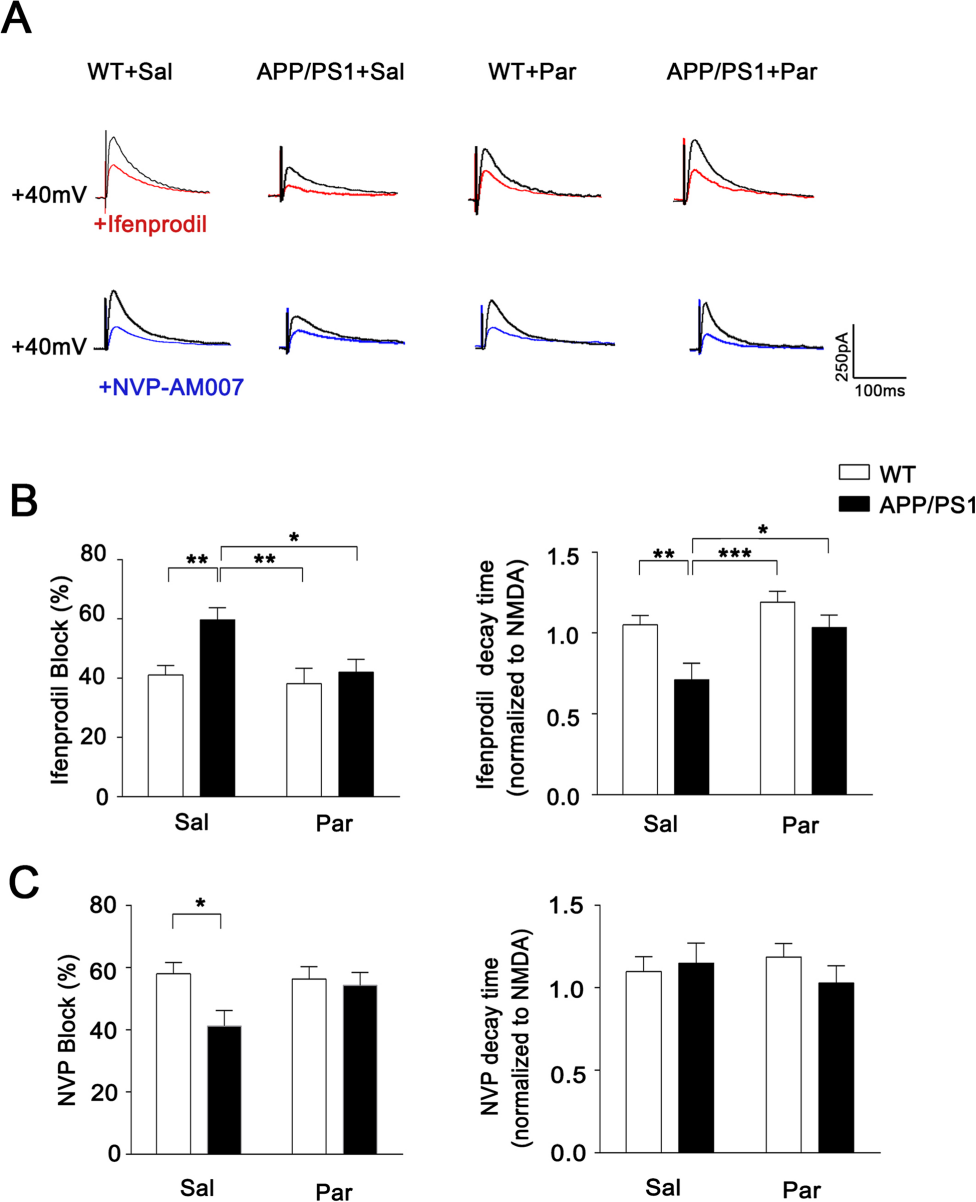
The GluN2A restoration in APP/PS1 after Paroxetine treatment according to NMDAR-mediated synaptic current. **a** Representative traces of NMDAR EPSC recorded at +40mv (black) and in the presence of ifenprodil (red) or NVP-AAM007 (blue). **b** Effect of ifenprodil application on NMDAR EPSC amplitude and decay time constant. **c** Effect of NVP application on NMDAR EPSC amplitude and decay time constant. Scale bar: 250pA (vertical) × 100 ms (horizontal). *n* = 12–20 neurons from 3 to 5 mice for per group. The data of decay time is normalized to the NMDA. Data are presented as mean ± SEM. * *P* < 0.05; ** *P* < 0.01; *** *P* < 0.001; two-way ANOVA with Sidak’s multiple comparison post hoc test

To evaluate the feature of particular NMDAR subunit contributing to NMDAR mediated EPSC, we measured the evoked NMDAR-EPSC amplitudes and decay times in the present of a GluN2B-specific antagonist ifenprodil or a GluN2A antagonist NVP respectively to discriminate GluN2A and GluN2B-medicated NMDAR currents [31, 32]. We observed that the peak amplitude of APP/PS1 mice after ifenprodil application was significantly decreased compared to WT mice, while the ifenprodil inhibitory effect in APP/PS1 mice was attenuated after long-term paroxetine treatment (Fig. 4a, b). In contrast, APP/PS1 mice showed the lack of sensitivity to NVP, exhibiting a decreased blocking rate in amplitude, which is compatible with the result of the reduced GluN2A expression in APP/PS1 mice (Fig. 4c). As the GluN2A-containing NMDARs have faster decay kinetics than GluN2B [33, 34], we further measured the decay time and observed that ifenprodil significantly shortened the current duration of APP/PS1 mice due to blocking long-time duration from GluN2B-containing NMDARs, while no change was seen in WT mice after ifenprodil application due to the less proportion of GluN2B-containing NMDAR than APP/PS1 mice. Therefore, the abnormal synaptic activity during development of cognitive defects in AD is attributable to the downregulation of GluN2A rather than GluN2B**.**

## Discussion

Our research found emotional dysfunction of young AD mice at 3 months that could be ameliorated through long-term paroxetine treatment, which would benefit for recognition function and preserved the eventual memory function in AD mice. Additionally, these neuroprotective effects of paroxetine are attributable to functional restoration of receptor activity of GluN2A-containing NMDAR in AD mice. Taken together, prophylactic administration of paroxetine could alleviate the AD progression by reducing Aβ levels and restoring GluN2A expression. This may provide appropriate intervention time in prodromal phases of clinical disease.

Previous findings have reported that Neuropsychiatric symptoms (NPS) are core features of Alzheimer’s disease and related dementias and these symptoms manifest commonly in very early disease and in prodromal phases [7, 35]. In our study, we **s**howed that AD mice displayed emotional dysfunction at 3 months age when cognitive function remains unaffected. Recently, several important studies have demonstrated that depression is a predisposing factor for the development of incident dementia [36–39]. Therefore, antidepressant use could potentially reduce risk for AD, particularly in subjects with abnormal emotional state and might be a potential therapy to prevent AD pathology. Although previous study show long-term paroxetine treatment did not improve anxiety-like activity and memory in old AD mice [5, 17], our findings indicates paroxetine treatment prophylactically in young AD mice ameliorates memory deficit, which was consistent with the effect of SSRIs in reducing risk of AD in depressed individuals from several clinical studies [40, 41].

Aβ accumulation and deposition in the AD brain can begin 10 years before the appearance of the first symptoms [42]. Several anti-Aβ therapeutic strategies are being pursued to treat AD. However, it is likely that treatment need to begin during the preclinical phase to prevent or limit plaque accumulation to be beneficial in reducing the risk of developing AD [43]. In our current study, treatment with paroxetine reduced Aβ levels. Similar effect of Aβ reductions by SSIRs has been reported previously.

The importance of NMDARs in synaptic plasticity and memory is well described. The replacement of GluN2B to GluN2A containing NMDARs has been demonstrated during postnatal development that is accompanied by synaptic maturation, stabilization and growth [44]. In contrast to GluN2B subunit, GluN2A subunit is predominantly present on large spines [44, 45]. In accordance with this, in our findings the GluN2A of APP/PS1 mice was largely reduced compared to WT mice and the reduction was eliminated by paroxetine treatment. The modulation of NMDARs may affect neural plasticity. Previous studies have found the effects of SSIRs on synaptic plasticity which was impaired in AD model [5], for example, fluoxetine has been found to facilitate LTP in the cerebral cortex, hippocampus and basolateral amygdala [23, 46]. Previous report has also examined the effect of long-term treatment with paroxetine on the neurogenic process in hippocampus, while no difference was observed in DCX^+^ (doublecortin) neuroblast number between WT and AD mice from postnatal 3  to 9 months, suggesting that the beneficial effects of Paroxetine may not due to neurogenesis [47].

In summary, we reveal the protective role of paroxetine in the early stage AD mice against initial memory decline. In light of the excellent safety record of paroxetine in long-term treatment of patients with depression and anxiety disorders, our findings suggest that intervention of paroxetine in human subjects for prodromal phase of AD are beneficial.

## Conclusions

We treated young adult APP/PS1 AD model mice prophylactically with paroxetine and investigated the protective role of anti-depressant agent in emotional and cognitive defects during disease progress. Our data indicate that prophylactic administration of paroxetine ameliorates the emotional dysfunction and memory deficit in AD mice. These neuroprotective effects are attributable to functional restoration of NMDAR subunit GluN2A in AD mice.

## Data Availability

All relevant data that support the findings of this study are available from the corresponding author upon reasonable request.

## Abbreviations

AD: Alzheimer’s disease
SSRI: Serotonin-selective re-uptake inhibitor
NPS: Neuropsychiatric symptoms
NFTs: Neurofibrillary tangles
MCI: Mild cognitive impairment
NSB: non-specific binding
BDNF: Brain-derived neurotrophic factor
PBS: Phosphate buffer saline
EPM: Elevated plus maze
ACSF: Artificial cerebrospinal fluid
CREB: cAMP response element-binding protein
DEGs: Differential expressed genes
LTP: Long-term potentiation
GCL: Granular cell layer
GO: Gene Ontology
WT: Wild-type
DAVID: Database for annotation, visualization and integrated discovery
NVP: NVP-AM007
DCX: Doublecortin
